# New Ultrasound Technologies for Ischemic Heart Disease Assessment and Monitoring in Cardiac Rehabilitation

**DOI:** 10.3390/jcm9103131

**Published:** 2020-09-28

**Authors:** Antonello D’Andrea, Simona Sperlongano, Mario Pacileo, Elio Venturini, Gabriella Iannuzzo, Marco Gentile, Rossella Sperlongano, Giuseppe Vitale, Marco Maglione, Gennaro Cice, Filippo Maria Sarullo, Anna Di Lorenzo, Carlo Vigorito, Francesco Giallauria, Eugenio Picano

**Affiliations:** 1 Unit of Cardiology, Department of Translational Medical Sciences, University of Campania “Luigi Vanvitelli”, Monaldi Hospital, 80131 Naples, Italy; sperlongano.simona@gmail.com; 2 Unit of Cardiology and Intensive Coronary Care, “Umberto I” Hospital, 84014 Nocera Inferiore (SA), Italy; m.pacileo@aslsalerno.it; 3 Cardiac Rehabilitation Unit, Azienda USL Toscana Nord-Ovest, Cecina Civil Hospital, 57023 Cecina (LI), Italy; vent.elio@tin.it; 4 Department of Clinical Medicine and Surgery, Federico II University, 80131 Naples, Italy; gabriella.iannuzzo@unina.it (G.I.); margenti@unina.it (M.G.); 5 Department of Experimental Sciences, University of Campania “Luigi Vanvitelli”, 80138 Naples, Italy; rossella.sperlongano@unicampania.it; 6 Cardiovascular Rehabilitation Unit, Buccheri La Ferla Fatebenefratelli Hospital, 90123 Palermo, Italy; giuseppevit@hotmail.com (G.V.); fsarullo@neomedia.it (F.M.S.); 7 Esaote, 50127 Florence, Italy; marco.maglione@esaote.com; 8 IRCCS San Raffaele Pisana, Via della Pisana 235, 00163 Roma, Italy; gennarocice@hotmail.com; 9 Department of Translational Medical Sciences, Federico II University of Naples, 80131 Naples, Italy; dilorenzoanna2@gmail.com (A.D.L.); vigorito@unina.it (C.V.); francesco.giallauria@unina.it (F.G.); 10 CNR Institute of Clinical Physiology Biomedicine Department, 56127 Pisa, Italy; picano@ifc.cnr.it

**Keywords:** ischemic heart disease (IHD), speckle tracking echocardiography (STE), global longitudinal strain (GLS), left atrial strain, tissue Doppler imaging (TDI), ventricular vortex, color Doppler flow mapping (CDFM), coronary flow reserve (CFR), arterial stiffness, cardiac rehabilitation

## Abstract

Owing to its ease of application, noninvasive nature, and safety, echocardiography is an essential imaging modality to assess cardiac function in patients affected by ischemic heart disease (IHD). Over the past few decades, we have witnessed a continuous series of evolutions in the ultrasound field that have led to the introduction of innovative echocardiographic modalities which allowed to better understand the morphofunctional abnormalities occurring in cardiovascular diseases. This article offers an overview of some of the newest echocardiographic modalities and their promising application in IHD diagnosis, risk stratification, management, and monitoring after cardiac rehabilitation.

## 1. Introduction

Ischemic heart disease (IHD) is the primary global cause of death. Mortality from IHD in Western countries has, however, dramatically decreased during the last decades owing to greater focus on primary prevention and improved diagnosis and treatment [1]. IHD diagnostic management includes the evaluation of patient’s symptoms and signs, the assessment of electrocardiogram, biochemical and echocardiographic findings, and further diagnostic tests (e.g., coronary computed tomography angiography, stress echocardiography, and single-photon emission computed tomography) if necessary. Once a diagnosis of obstructive coronary artery disease (CAD) has been confirmed, the patient’s event risk will be determined, and the appropriate therapy will be chosen [2]. In this context, rest and stress echocardiography play a central role in supporting diagnosis and risk stratification of IHD, providing information about myocardial systolic global and regional function of both left and right ventricle. Transthoracic echocardiography (TTE), because of its ease of application, noninvasive nature, and safety, is the method of choice for assessment of cardiac structure and function. Besides diagnosis, TTE is a useful tool in guiding therapeutic decision-making and in monitoring response to therapy.

Over the last decades, several technological advancements were made in the echocardiographic field, aiming to better understand the morphofunctional abnormalities occurring in cardiovascular diseases. These technologies are still struggling to enter the daily and routine use. This review wants to turn the spotlight on some of the newest cardiac ultrasound imaging technologies highlighting their promising application to the IHD diagnosis, management, and monitoring during cardiac rehabilitation.

## 2. Left Ventricular Strain

During ejection, left ventricular (LV) myocardium undergoes a three-dimensional (3D) deformation, characterized by longitudinal and circumferential shortening, and radial thickening. Added to this is the rotation of the LV around its long axis, i.e., viewed from apex, the apex rotates in anticlockwise direction, whereas the base rotates in the clockwise direction. The movement caused by this opposite rotation is called twist and is crucial for the LV ejection performance. The untwist, which occurs during diastole, generates a suction force that drives the early, rapid diastolic filling of the LV [3]. This complex multi-dimensional deformation during the cardiac cycle is possible owing to the double-helical orientation of LV myocardial fibers, i.e., endocardial helix is more parallel to the LV long axis and is associated primarily with the longitudinal deformation, whereas epicardial helix is responsible primarily for the circumferential shortening. Both subendocardial and subepicardial fibers contribute to radial thickening and to LV rotational movement.

Two-dimensional (2D) speckle tracking echocardiography (STE) is a gray-scale based technique of myocardial deformation imaging, independent from insonation angle, which allows to assess all the above-mentioned components of LV contractile function. The myocardial deformation is quantified as strain or strain rate (SR). Strain is the percentage change in the length of a myocardial segment compared to its resting length; SR is the rate at which this deformation takes place, expressed as 1/s. Normally, strain and SR have negative values in systole when myocardium shortens and positive values in diastole when myocardium lengthens. The STE software identifies a number of bright speckles generated by the scatter of the ultrasound beam after its interaction with the myocardium and follows them frame-by-frame. Then, through an algorithm, it calculates the magnitude of myocardial deformation in each direction and generates strain and SR curves. The strain analysis is performed on a workstation where gray-scale images are transferred as digital media. Anyway, some of the currently available ultrasound systems offer the possibility of online STE analysis, on the echocardiographic machine itself, even on portable devices.

In order to calculate the LV longitudinal strain, images of high quality from the apical 4-chamber (4CH), 2-chamber (2CH), and long-axis (LAX) view should be taken, so that the LV occupies most of the sector and is not foreshortened. The optimal gray-scale frame rate should be kept between 30 and 70 frames/s. Three cardiac cycles should be acquired for each view (ECG-gating is mandatory). The images can be transferred to the workstation. It is recommended to start the analysis from the apical long-axis view, where the aortic valve leaflets motion helps to identify the timing of valve closure, that is essential in the deformation study. The endocardial borders are manually traced in the end-systolic frame automatically brought up by the software. A region of interest (ROI), including the entire myocardial thickness, is generated by the software and can be manually modified in width. The software then tracks the myocardial speckles frame-by-frame, producing a moving image displaying the tracking during the cardiac cycle. If the tracking seems to be not adequate, the operator can go back and adjust the ROI or select a new ROI. Once the tracking is approved by the operator, the software divides the LV myocardium of the view in analysis in 6 segments, providing segmental and global longitudinal strain, myocardial velocities, and strain curves. The same process must be repeated for the apical 4-chamber and 2-chamber views to obtain strain values for all myocardial segments, their average, and the LV global longitudinal strain (GLS). Some ultrasound systems also provide the bull’s eye, which intuitively displays segmental and global peak-systolic longitudinal strain.

A right ventricular (RV) focused 4-chamber view is required to analyze RV strain, whereas short-axis views are required to obtain information about circumferential and radial deformation (basal, mid, and apical levels) and LV twist (basal and apical levels).

**Figure 1:**
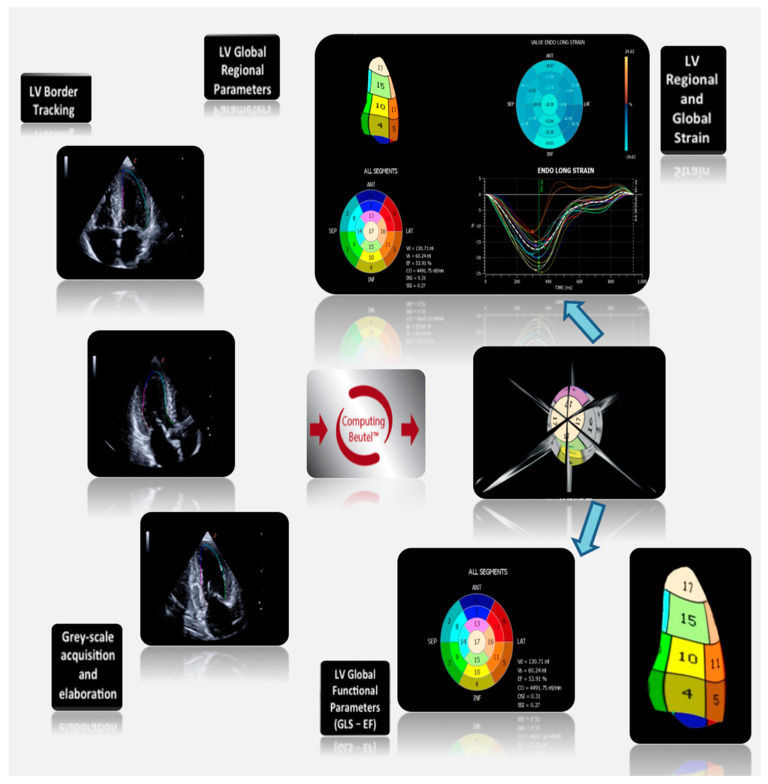
XStrain 4D global LV analysis. At the end of each scanning section, the three apical views are acquired. Then, after left ventricular (LV) endocardial border tracking, the software analyzes LV regional deformation parameters. Finally, the Beutel 3D reconstruction allows quantification of global LV function (global longitudinal strain (GLS)—ejection fraction). XStrain^TM^ 4D, done with MyLab X8 eXP—Esaote S.p.A., Florence, Italy.

**Figure 2:**
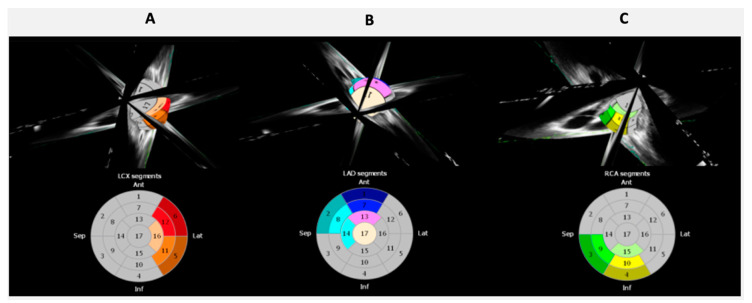
XStrain 4D regional LV analysis. Left ventricular (LV) regional deformation parameters in three coronary perfusion regions: left circumflex (**A**), left anterior descending (**B**), and right coronary artery (**C**).

Recently, 3D STE has been introduced by applying speckle tracking technologies to 3D echocardiographic images. Images are usually acquired using a matrix-array transducer, from the apical position in a wide-angled acquisition ‘‘full-volume’’ mode. In this mode, a number of wedge-shaped subvolumes are acquired over consecutive cardiac cycles during a single breath hold and stitched together to create one pyramidal volume sample. A major limitation of 3D STE to date is the temporal resolution of the volumetric pyramidal data sets. Usually the rate of acquisition does not exceed 20–30 volumes/s, and, in most cases, to obtain higher temporal resolution, the field of view needs to be considerably narrowed. By fusing 2D speckle tracking information obtained from standard apical 4CH, 2CH, and LAX views, XStrain™ four-dimensional (4D) aims to make myocardial quantification imaging interpretation easier by the 3D/4D reconstruction of the LV. The user can freely rotate and zoom the beutel and superimpose the echographic scanning planes to better evaluate the contractility properties of the LV, using a physiological tool to analyze the complex multi-dimensional LV mechanics [4], including a parallel assessment of myocardial regional and global function (Figure 1). By this technique, it is possible to focus the strain analysis on single coronary artery supply regions (Figure 2), allowing better correlations between visual assessment of regional wall motion and quantification of regional deformation.

### Role of LV Strain in IHD

STE is able to detect subclinical LV systolic dysfunction in an early-stage cardiovascular diseases, when LV ejection fraction (LVEF) is still normal. Actually, an isolated impairment of one myocardial layer can be present, compensated by the augmentation of function of the other layers, so that LVEF and overall LV performance remain preserved [5]. For example, in subendocardial ischemia, where there’s an involvement of subendocardial fibers, the reduced longitudinal deformation can be compensated by increase in circumferential strain and twist. On the other side, patients with myocardial transmural involvement due to transmural infarction show impairment of both longitudinal and circumferential strain, fall in LVEF, and dilatation of LV cavity.

Strain and SR parameters are significantly reduced in ischemic-segments in patients with acute myocardial infarction (AMI) [6]. Regional longitudinal strain is markedly lower in areas with LGE on cardiac magnetic resonance (CMR) than the normal myocardium, and it gives information on the transmural extent of necrosis [6,7]. A cutoff value of −11.5% identified the transmural-infarcted segments with a sensitivity of 75% and a specificity of 78% in a study by Cimino et al. [7].

Multilayer longitudinal strain assessed by 2DSTE, in particular endocardial strain, is useful to identify patients with AMI and significant/complex CAD [8,9,10].

As for the LV, RV peak systolic longitudinal strain is able to predict RV scar and well correlates with scar extent [11].

STE adds a prognostic value to the traditional indexes of LV systolic function in both stable and intensive care setting [12]. Longitudinal strain parameters revealed to be independent predictors of death and cardiovascular complications after AMI [13,14,15].

Peak systolic GLS was also identified as independent predictor of LV function recovery during the follow-up of 12 months after AMI [16]. A low GLS (≤12%) after PCI in a population of patients with recent NSTEMI was predictor of negative LV remodeling at follow-up [17,18].

Finally, in a population of patients with ischemic dilated cardiomyopathy, D’Andrea et al. showed that GLS was strongly correlated with the total scar burden assessed by CMR (r = 0.64, *p* < 0.001), and was an excellent independent predictor of response to resynchronization therapy (−10.4% in nonresponders vs. −18.4% in responders, *p* < 0.001) [19].

## 3. Left Atrial Strain

The main role of left atrium (LA) is to modulate LV filling and cardiovascular performance. For this purpose, LA works as reservoir during ventricular systole, when it receives blood from pulmonary veins and its volume increases, as conduit during early ventricular diastole, passively allowing the passage of blood into the ventricle, and as booster pump during late ventricular diastole, when it actively contracts to complete LV filling. During last years the application of 2DSTE to the LA has been largely investigated in several studies [20,21,22,23,24,25,26,27,28]. Longitudinal strain and SR curves are generated for each of the 6 atrial segments, obtained from apical 4- and 2-chamber views. Anyway, atrial curves show an opposite morphology than ventricular curves, since that atria and ventricles move in opposite directions during the cardiac cycle. Analogous to the concept of LVGLS, where longitudinal systolic myocardial deformation is quantified, peak atrial longitudinal strain (PALS) detects the maximum elongation of the LA during LV systole, at the end of the reservoir phase. The major determinant of LA expansion is the downward displacement of the LV base toward the apex during LV systole. Therefore, any condition with adverse effect on the LV longitudinal myocardial function will also reduce PALS. Strain is a measure of deformation and is, therefore, dependent on the baseline LA length. Severe LA wall remodeling, like in long-standing atrial fibrillation, leads to progressive LA fibrosis and dilatation, with reduced PALS. The mean value of PALS in healthy subjects is 42%, for LVGLS around −21%.

### Role of LA Strain in IHD

LA reservoir and conduit functions, respectively, evaluated during systole and early diastole by 2DSTE, are impaired in patients with CAD, even in absence of LA enlargement [29]. LA reservoir dysfunction may reflect a diastolic dysfunction of the LA, likely caused by ischemia. LA conduit dysfunction may be, rather, due to ischemia-related LV compliance impairment during diastole. In patients with CAD and LA enlargement, even LA booster pump function, assessed during late diastole, is significantly impaired, likely due to several factors, such as LV compliance alteration, LV filling pressures elevation, LV systolic dysfunction, and LA myocardial injury. These findings may prove that LA diastolic dysfunction occurs prior to LA systolic dysfunction in patients affected by IHD.

LA strain and SR could be useful in predicting the severity of CAD. Actually, a strong inverse correlation between atrial deformation parameters and severity of coronary stenosis was found [30]. Moreover, LA booster pump function is enhanced in patients with left anterior descending (LAD) occlusion, despite the impairment of reservoir function, probably as response to the increased afterload due to the stiff LV. Conversely, patients with proximal circumflex (Cx) occlusion show LA contractile dysfunction, as the LA is perfused by branches arising from the proximal Cx [30,31].

## 4. Tissue Doppler Imaging (TDI)

TDI is an echocardiographic technique which allows to measure myocardial velocities using Doppler principles. TDI can be performed in three different modalities: pulsed wave (PW), color, and 3D mode [32]. Pulsed wave tissue Doppler imaging (PWTDI) measures the instantaneous regional myocardial peak velocities. A sample volume of 5–7 mm is placed in the ventricular myocardium, adjacent to the mitral annulus, in apical views, in order to obtain longitudinal myocardial velocities which are a good surrogate of LV longitudinal contraction and relaxation. PWTDI has high temporal resolution but does not permit the simultaneous analysis of more myocardial segments. With color TDI, a color-coded box is superimposed to gray-scale 2D or M-mode images, indicating myocardium direction and velocity. Color TDI has higher spatial resolution, it allows simultaneous interrogation of the entire color box, but needs offline quantification of myocardial velocities. Offline velocities obtained by color TDI are approximately 20–25% lower than those obtained from PWTDI. With 3D TDI, a color-coded TDI is applied to the triplane apical view (simultaneous acquisition of 4-, 2-, and 3-chamber view). Velocities’ analysis is performed offline. 3D TDI also allows to calculate LV volumes and EF.

PWTDI represents the cardiac cycle throughout three waveforms: systolic myocardial velocity (S’) above the baseline, early diastolic myocardial relaxation velocity (e’) below the baseline, and late diastolic myocardial velocity associated to atrial contraction (a’) below the baseline. Systolic and diastolic time intervals can be obtained using TDI, such as isovolumic contraction time (IVCT), ejection time (ET), and isovolumic relaxation time (IVRT). Finally, the myocardial performance index (MPI) can be calculated as the sum of IVCT and IVRT divided by the ET.

### Role of TDI in IHD

A significant decrease of S’, e’, and a’ waves has been observed in patients with IHD, even if cutoff values for maximum systolic and diastolic velocities which detect CAD are still unclear [33].

S’ velocity can be used to quantify the entity of regional motion impairment. In a systematic review by Agarwal et al., the authors suggest that S’ velocity may be of help in detecting CAD especially in patients with poor 2D endocardial definition, where the qualitative wall motion analysis can be difficult.

During both acute and chronic ischemia, the phenomenon of postsystolic shortening (PSS) can be quantified by TDI or STE. It is a myocardial shortening that takes place after aortic valve closure and therefore does not contribute to LV ejection. This phenomenon is probably due to the passive recoil from the interaction between ischemic and surrounding nonischemic myocardial segments. Several studies proved that PSS indicates myocardial viability and it is a predictor of systolic recovery in patients with IHD [34,35,36]. Anyway, data in literature are still conflicting. Terkelsen et al., who assessed PSS by TDI in a STEMI population after revascularization, concluded that PSS was not a marker of cardiac viability because it did not correlate with improvement in strain or wall motion score index (WMSI) at follow-up [37]. Moreover, Brainin et al. found that PSS was an independent predictor of HF in patients following ST-elevation myocardial infarction (STEMI) [38].

## 5. Color Doppler Flow Mapping

Analysis of intracardiac flows represents a way to approach the study of LV function [39]. Normally, during diastole, when blood flow enters the LV from the LA, it gives rise to the formation of two vortices, which rotates around a virtual axis, storing kinetic energy: a main, anterior vortex, which rotates clockwise, and a secondary, posterior vortex, which rotates counterclockwise. The main vortices’ determinant is the natural asymmetric geometry of the mitral valve apparatus (the anterior leaflet is longer than the posterior, and the mitral valve orifice is eccentric as compared with the LV axis).

**Figure 3:**
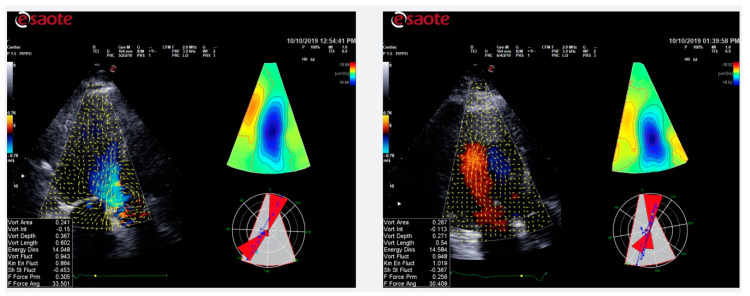
LV vortex analysis. During diastasis, left ventricular (LV) vortex maintains its rotary motion. Subsequently, at the moment of atrial contraction (late filling), a second vortex ring occurs, and the late filling jet combines with the residual early vortex ring. During the isovolumic contraction period, the vortex redirects blood toward the LV outflow tract, with formation of a large anterior vortex across the inflow–outflow region. HyperDoppler software by Esaote S.p.A on MyLab X8 eXP scanner.

Even if phase-contrast magnetic resonance imaging is the gold standard for measuring blood velocities in heart cavities, some echocardiographic techniques have been developed in order to visualize intracardiac flow, among which is the color Doppler flow mapping (CDFM). The HyperDoppler software is a recent CDFM-based technology that provides different possibilities to represent intracardiac flow data: a flow velocity vector map where velocity vectors are displayed as arrows superimposed on the traditional color Doppler flow images which can be followed frame by frame; a circulation parametric map where vortices are represented as compacted regions in blue (clockwise rotation) or in red (counterclockwise rotation); and a kinetic energy map where the highest level of kinetic energy is depicted in red. Normally, intracardiac vortices are visualized on apical long-axis view images (Figure 3). 

### Role of CDFM in IHD

The LV vortex formation is the result of an optimal interaction between LV chamber geometry, morphology of the mitral valve apparatus, and normal electrical conduction system, which allows the harmonic contraction of the cardiac walls [39]. If one of these elements is altered, the LV vortex formation is affected too.

In patients with STEMI, kinetic energy dissipation within the LV increases linearly with the increase of LVEF, as the flow turbulence into the LV is higher [40,41]. For the same reason, larger infarctions are associated with a more severe alteration in LV intracavitary blood flow dynamics and a lower dissipation in kinetic energy [42]. 

Finally, in patients with anterior myocardial infarction, LV vortex flow analysis was useful to identify patients at risk for LV apical thrombus formation [43]. 

## 6. Coronary Flow Reserve

**Figure 4:**
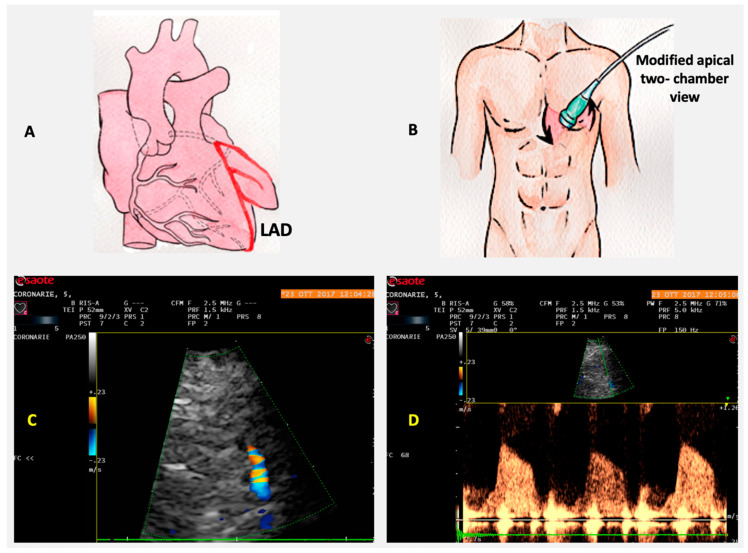
Coronary flow analysis. The distal left anterior descending (LAD) tract is more suitable to investigate coronary microvascular function because it is between large epicardial arteries and microvasculature (**A**). The acoustic window is, in the left decubitus position, around the midclavicular line in the fourth or fifth intercostal space (**B**). After detecting by color the LAD image (**C**), coronary flow by Doppler is represented by a biphasic wave, with a lower peak during systole and a higher peak during diastole, for the effect of myocardial contraction (**D**).

The evaluation of coronary flow reserve (CFR) is performed by combining transthoracic Doppler echocardiography with vasodilator stress, such as adenosine or dipyridamole. An index of CFR is obtained from the variation between coronary blood flow velocity (CBFV) at baseline and CBFV at the peak of effect of vasodilator. A value of CFR less than 2 is generally considered abnormal. CBFV profile is recorded by PW Doppler, and it is represented by a biphasic wave, with a lower peak during systole and a higher peak during diastole. This pattern derives from the fact that myocardial extravascular resistance is higher in systole, due to the effect of myocardial contraction. The most used parameter in evaluation of CFR is peak diastolic flow, because it is easy to measure, reproducible, and has a closer correlation with CFR measured by Doppler flow wire and positron emission tomography (PET) [44]. The LAD peak diastolic flow is obtained in the mid-distal portion of the coronary artery, from a modified apical 2CH view, where the transthoracic probe is slightly moved upward and medially, with a little counterclockwise rotation and medial angulation. CFR feasibility of LAD is very high, reaching 98%, and the use of contrast is rarely needed, when the Doppler signal is not appropriate (Figure 4). Noninvasive CFR evaluation is also possible for posterior descending artery (PDA) and Cx, even if with a lower technical feasibility. PDA is assessed from a modified apical 2CH view showing the posterior interventricular groove, near to coronary sinus ostium. Cx artery is searched in apical 4CH view at basal and midportion of LV lateral wall, and it is the most challenging to evaluate, due to its particular anatomy and the low resolution of the lateral wall. Contrast use may help to increase feasibility of CFR in PDA and Cx. Finally, transthoracic Doppler echocardiography may be useful in evaluation of coronary artery bypass graft (CABG) patency, sampling the coronary flow downstream of graft anastomosis.

### Role of CFR in IHD

Stress echocardiography was recently reshaped with the ABCDE protocol: A for asynergy, B for thoracic ultrasound B-lines, C for contractile reserve, D for Doppler-based CFR (in LAD), and E for electrocardiogram-based heart rate reserve (HRR, defined as peak/rest HR  <  1.62) [45]. ABCDE protocol allows therefore a comprehensive assessment focused on ischemia (A), pulmonary congestion (B), myocardial scar or necrosis (C), coronary microvascular dysfunction (D), and chronotropic incompetence (E) [46]. 

CFR is a composite measure of coronary macrovascular and microvascular status. A reduced CFR is associated to a negative prognosis in several groups of patients, including those with diabetes mellitus, arterial hypertension, ischemic cardiomyopathy, normal coronaries, and CAD [47]. In patients with IHD, CFR is related to angiographic findings and it is a predictor of the extent and severity of CAD [48]. 

Recently, the assessment of CFR on TTE has been proposed as method to detect cardiac allograft macro- and microvasculopathy in heart transplant [49]. A recent study showed that CFR is very sensitive for detecting cardiac allograft vasculopathy and increases the diagnostic accuracy of dobutamine stress echocardiography [50]. 

## 7. Arterial Stiffness

Vascular aging in large arteries is characterized by structural and functional changes, such as intima-media thickening and “stiffening” [51]. Arterial wall thickening is mainly related to atherosclerotic processes; arterial stiffening, which is the vessel wall’s tendency to resist deformation generated by systolic blood pressure during the cardiac cycle, is mainly due to degenerative and calcified processes.

**Figure 5:**
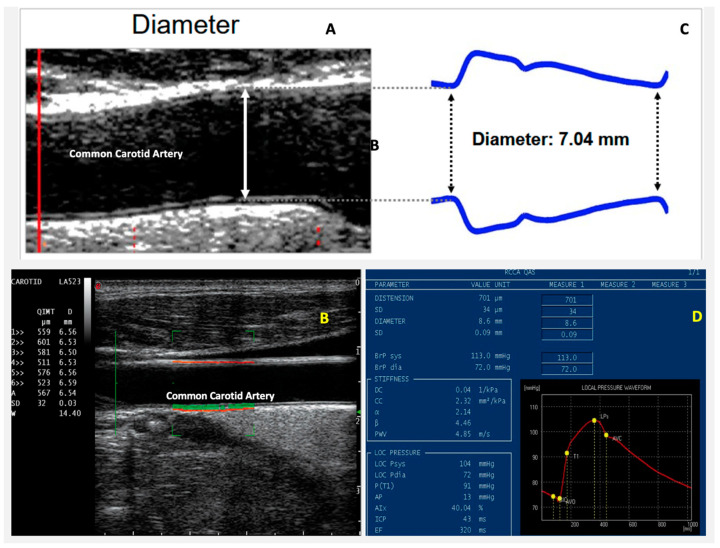
Vascular morphologic and functional analysis. Radio Frequency-Quality Intima Media Thickness (^RF^QIMT): during the scanning of the carotid artery a real-time feedback on measurement quality via quality indicators overlaid on the ultrasound image at the position of the vessel wall (orange lines) and the far wall intima layer (green line) (**A**,**B**). Radio Frequency Quality Arterial Stiffness (^RF^QAS) targets the measurement of the blood vessel stiffness of a subject in a selected area of investigation. The blood vessel wall stiffness is expressed as pulse wave velocity obtained from brachial blood pressure and the accurate measurements of diameter and change in diameter. Moreover, the local blood pressure at the site of the ultrasound measurement is given (**C**,**D**). ^RF^QIMT and ^RF^QAS, respectively—Esaote.

The recently introduced radiofrequency (RF) data technology allows to measure arterial intima-media wall thickness (IMT) and stiffness. All measurements are taken in a selected area of common carotid artery. The blood vessel wall stiffness is expressed in pulse wave velocity (PWV) in meter per second, and is obtained from the brachial blood pressure and the accurate measurements of vessel diameter and distension (change in diameter). For both IMT and stiffness, the operator gets real-time feedback on measurement quality via quality indicators overlaid on the ultrasound image. This real-time feedback gives the operator the possibility to optimize his probe position, in order to obtain a scan plane perpendicular to the wall of the common carotid artery (Figure 5).

### Role of Arterial Stiffness in IHD

Vascular intima-media thickening and stiffening are correlated to cardiovascular morbidity and mortality [50]. Arterial stiffness plays an important role in systolic blood pressure (BP) and pulse pressure (systolic BP—diastolic BP) increase. Moreover, it can contribute to LV concentric remodeling and hypertrophy, impaired myocardial perfusion, and other changes in LV that form the substrate for systolic and diastolic dysfunction. 

PWV is closely linked to the presence of angiographically documented CAD [52]. Moreover, elevated PWV values are associated to an increased risk in cardiovascular events in both patients with and without pre-existing CAD [53,54,55,56]. 

In the EPHESUS study, performed on patients with heart failure and reduced LVEF following AMI, it was shown that an increased aortic stiffness, assessed by PWV, was associated with a negative prognosis and significantly contributed to cardiovascular death [57].

## 8. Advantages and Limitations of the New Ultrasound Technologies

**Figure 6:**
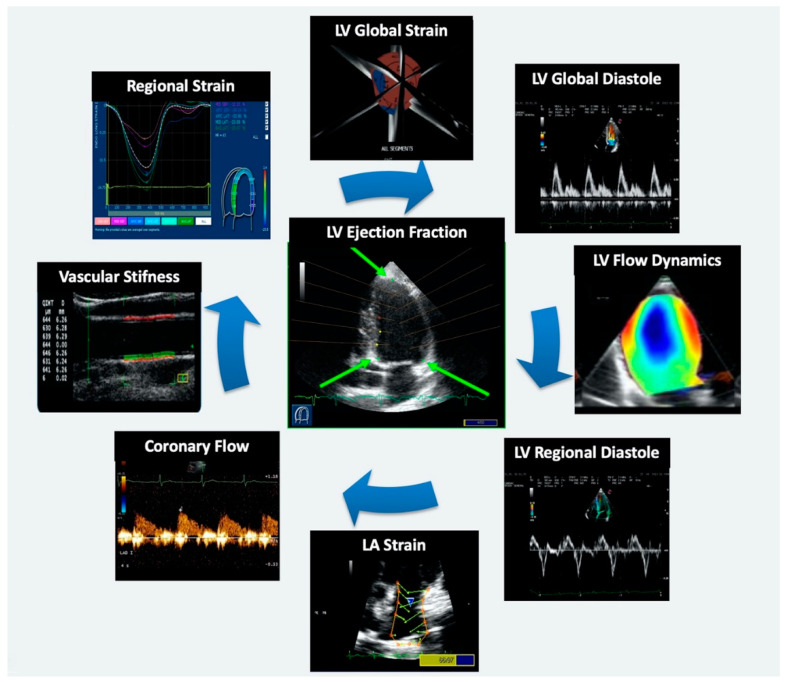
From standard echocardiography to new technologies. The incremental contribution of new echo technologies to provide valuable insights in the pathogenesis of cardiovascular disease, with distinct patterns of myocardial, vascular, and microcirculatory dysfunction. LV: left ventricle. LA: left atrial.

All the above-mentioned echocardiographic technologies have the advantage of being safe, noninvasive, and relatively low cost, and they can be performed bedside. Moreover, they allow to better understand the morphofunctional abnormalities occurring in cardiovascular diseases (Figure 6). Table 1 summarizes specific advantages and limitations of each new technology described in this review.

## 9. Cardiac Rehabilitation and New Echocardiographic Technologies

Exercise-based cardiac rehabilitation (CR) in patients affected by CAD increases functional capacity and reduces total and cardiovascular morbidity and mortality [58,59,60,61,62]. A role in this improvement induced by exercise, may be played by reverse LV remodeling characterized by reduced LV volumes and increased LV ejection [63]. Echocardiography is a pivotal tool to evaluate LV stroke volume (SV), cardiac output (CO), and EF, even compared to other noninvasive techniques. A recent study by Gonzalez-Represas and Mourot proved that tonometry and impedancemetry overestimate absolute SV and CO values when compared to echo, and they are not useful to reliably track changes of these parameters in CAD patients after a CR program [64]. Traditional echocardiographic indexes should be desirably integrated with the ones deriving from new ultrasound technology. For instance, LVEF alone does not well define LV global systolic function, it is less sensitive for the detection of subclinical contractility changes, and it does not allow every time to recognize the passive motion of myocardial segments which are dragged by adjacent normal segments. The assessment of LV mechanics, with its 3D deformation and rotation, through STE may help to better quantify the functional LV reverse remodeling after MI. McGregor et al. showed that LV twist and twist velocity were significantly reduced in patients with MI who underwent CR exercise training than nonexercised patients, data which may indicate increased systolic efficiency, greater “twist reserve,” and so, a favorable adaptation of the LV compromised by MI [65]. GLS was unaffected by exercise training in the study carried out by McGregor et al. Conversely, other studies showed that GLS and radial strain improved in patients who underwent exercise training after MI when compared to patients with MI and without CR [66,67]. Post-MI CR seems to have favorable effects also on LA function evaluated by STE [68]. The positive LA remodeling induced by CR may protect patients from atrial arrhythmias, which increase the risk of stroke and all cause of mortality after acute MI.

Data regarding the clinical application of intracardiac flow analysis to patients who undergo CR after MI are still missing in literature. It would be attractive to evaluate LV vortex features and kinetic energy dissipation before and after a CR program, in order to obtain a further index of LV remodeling and function.

An interesting recent pilot study explored the effects of standard CR with and without transcendental meditation (TM) on CFR assessed by ^13^N-ammonia PET. For the combined TM group, CFR increased when compared to the combined non-TM group [69]. These results may be applied to the design of controlled clinical trials to definitively test these effects also on CFR assessed by echocardiography.

Even arterial stiffness evaluation finds an interesting application in CR field. In particular, PWV was shown to be decreased in absence of changes of peripheric arterial blood pressure in patients with IHD undergoing a CR program [70,71,72]. Moreover, a “dose–response” association between the reduction of arterial PWV and the number of CR sessions was observed. The improved ventricular-vascular coupling derived from reduced arterial stiffness could contribute to the better functional capacity and the lower cardiovascular morbidity of patients who undergo CR.

Table 2 illustrates the most commonly collected traditional echocardiographic parameters and the measurements deriving from new ultrasound technologies, which may be assessed before and after a program of CR.

## 10. Conclusions

Standard echocardiography is an essential imaging modality for the assessment of patients affected by IHD, due to its unmatched ability to combine safety and ease of application with depth of diagnostic and prognostic information. The newer ultrasound technologies, which were developed over the past few decades, allowed to better understand the morphofunctional abnormalities occurring in cardiovascular diseases. These technological advancements revealed to be promising to further expand the role of echocardiography as modality of choice in IHD diagnosis, risk stratification, management, and monitoring after cardiac rehabilitation.

## Figures and Tables

**Table 1:** Advantages and limitations of the new ultrasound technologies.

Technology	Advantages	Limitations
Speckle tracking echocardiography	- Less angle-dependency than TDI - Study of deformation in any direction - Evaluation of regional and global function in short time - Low noise and artifacts interference - Good reproducibility - Semiautomated processing (less operator dependent) - Possibility of analysis of right ventricle and atria deformation	- Image quality dependency - Frame rate dependency - Low frame rate (limited temporal resolution) - Tracking affected by out of plane cardiac motion - Intermachine and inter-software variability - Afterload, preload, and heart rate dependency - More variability of radial and circumferential strain than longitudinal strain measurements
Tissue Doppler imaging	- Excellent temporal resolution - Online analysis of velocities	- High angle dependency - Size and placement of sample volume done manually - Deformation assessment in one direction - Noise interference - Global heart motion influence
Color Doppler flow mapping	- Good spatial 2D resolution - Good time resolution - Accuracy in evaluation of high flow velocity - Contrast medium not required - Short processing time	- Underestimation of low flow velocity - Reconstruction of velocities perpendicular to beam direction - Acoustic window dependency - Nonvalidated vs. the gold standard (magnetic resonance)
Coronary flow reserve	- High accuracy and reproducibility for LAD - Contrast medium rarely needed for LAD - Combined assessment of coronary flow and wall motion	- Limited sites of coronary detection - Lower feasibility for posterior interventricular artery and circumflex artery - Angle dependency - Artifacts interference
Carotid stiffness	- Good accuracy and reproducibility - Early detection of cardiovascular damage	- Limited diffusion - Few studies on large populations

TDI: tissue doppler imaging; LAD: left anterior descending; 2D: Two-dimensional.

**Table 2:** Traditional and new echocardiographic measurements to assess before and after a CR program.

Category	Traditional Indexes	New Technologies Indexes
LV dimension and systolic function	- LVEDD, LVESD - LVEDV, LVESV - LVEF - -SVi, COi	- Septal and lateral S’ waves - GLS - Radial strain, circumferential strain, twist - Vortex properties
LV diastolic function	- E and A waves - E/A ratio - E wave DT	- Lateral and septal e’ waves - Average E/e’ ratio
LA dimension and function	- AP diameter - LAVi	- LA strain
RV dimension and systolic function	- RVD (basal, mid-cavity, longitudinal) - TAPSE - FAC	- S’ wave - GLS
Arterial stiffness	/	- PWV

CR: cardiac rehabilitation; LV: left ventricular; LVEDD: left ventricular end diastolic diameter; LVESD: left ventricular end systolic diameter; LVEDV: left ventricular end diastolic volume; LVESV: left ventricular end systolic volume; LVEF: left ventricular ejection fraction; SVi: stroke volume index; COi: cardiac output index; GLS: global longitudinal strain; DT: deceleration time; LA: left atrial; AP: antero-posterior; LAVi: left atrial volume index; RV: right ventricular; RVD: right ventricular diameter; TAPSE: tricuspid annulus plane systolic excursion; FAC: fractional area change; PWV: pulsed wave velocity.
